# Elevated tumor and serum levels of the hypoxia-associated protein osteopontin are associated with prognosis for soft tissue sarcoma patients

**DOI:** 10.1186/1471-2407-10-132

**Published:** 2010-04-08

**Authors:** Matthias Bache, Matthias Kappler, Henri Wichmann, Swetlana Rot, Antje Hahnel, Thomas Greither, Harun M Said, Matthias Kotzsch, Peter Würl, Helge Taubert, Dirk Vordermark

**Affiliations:** 1 Department of Radiotherapy, Martin-Luther-University Halle-Wittenberg, Halle, Germany; 2 Department of Oral and Maxillofacial Plastic Surgery, Martin-Luther-University Halle-Wittenberg, Halle, Germany; 3 Radiation Oncology, University of Würzburg, Würzburg, Germany; 4 Institute of Pathology, Dresden University of Technology, Dresden, Germany; 5 Department of Surgery, Malteser St Franziskus Hospital gGmbH, Flensburg, Germany

## Abstract

**Background:**

Osteopontin (OPN) overexpression is correlated with a poor prognosis for tumor patients. However, only a few studies investigated the prognostic impact of expression of OPN in soft tissue sarcomas (STS) yet.

**Methods:**

This study is based on tumor and serum samples from 93 adult STS patients. We investigated OPN protein levels in serum (n = 86) and tumor tissue (n = 80) by ELISA and OPN mRNA levels in tumor tissue (n = 68) by quantitative real-time PCR.

**Results:**

No correlation was found between OPN levels in serum and tumor tissue. Moreover, an elevated OPN protein level in the serum was significantly associated with clinical parameters such as higher stage (p = 0.004), higher grade (p = 0.003), subtype (p = 0.002) and larger tumor size (p = 0.03). OPN protein levels in the tumor tissue were associated with higher stage (p = 0.06), higher grade (p = 0.003), subtype (p = 0.07) and an increased rate of relapse (p = 0.02). In addition, using a Cox's proportional hazards regression model, we found that an elevated OPN protein level in the serum and tumor tissue extracts is a significant negative prognostic factor for patients with STS. The relative risks of tumor-related death were 2.2 (p < 0.05) and 3.7 (p = 0.01), respectively.

**Conclusion:**

Our data suggest OPN protein in serum as well as in tumor tissue extracts is an important prognostic factor for soft tissue sarcoma patients.

## Background

OPN, a phosphorylated glycoprotein, is detected in body fluids as well as in tumor tissues. Initially, it was suggested that OPN is expressed by macrophages infiltrating the tumor rather than by tumor cells themselves [1]. However, elevated serum or plasma levels of OPN have been detected in a variety of human cancers, which has been correlated with tumor progression and metastasis [2-4]. Moreover, tumor OPN protein over expression is also linked to a worse prognosis in different tumor entities such as breast cancer [5,6], lung cancer [7], cervical cancer [8,9], prostate cancer [10] and head and neck cancer [11-13]. Furthermore, previous studies suggest that high tumor OPN mRNA expression also correlates with an unfavorable prognosis [14-18].

In support of its prognostic value, direct or indirect inhibition of the OPN signaling pathway results in reduced malignant tumor potential with effects on cell survival, migration, invasion, tumor growth and metastasis (reviewed in [4,19,20]). However, only a few studies have investigated the role of osteopontin in tumor progression of sarcoma patients. For example, in primary sarcomas of the pulmonary artery, OPN protein could be detected in tumor cells and in macrophages and it is potentially involved in tumor progression and metastasis [21]. A further immunohistochemical study of STS showed that OPN in tumor tissue is associated with tumor stage, grade and overall survival of STS patients at 5-years [22]. However, another study using immunohistochemical evaluation of OPN expression did not provide predictive information on the outcome of osteosarcoma patients [23]. We therefore analyzed OPN mRNA and protein levels of tumor samples and OPN serum levels of 93 histological verified adult STS patients. Moreover, to characterize the role of OPN in soft tissue sarcoma, we correlated OPN levels with clinical parameters and prognosis.

## Methods

### Patients and tumor material

Tumor tissue and serum samples of 93 adult patients with histologically verified primary STS were analyzed (Table 1). In detail, we have analyzed OPN protein levels in serum (86 patients) and tumor tissue (80 patients) and OPN mRNA level in tumor tissue (68 patients). Patients and tissue samples have been partially described previously [24-26]. The study was carried out in compliance with the Helsinki Declaration and it was approved by the Ethics Committee of the Medical Faculty of the University Halle. All patients gave written informed consent (Institute of Pathology, University of Halle, Germany and Department of Surgery 1, University of Leipzig, Germany). Patients' mean age was 56 years (19 to 87 years). The median follow-up time was 44 months (2 to 146 months). The overall survival according to stage was 86% for stage I (n = 15), 59% for stage II (n = 39), 37% for stage III (n = 19) and 0% for stage IVa (n = 9). Tumor tissue was collected following surgical resection and snap-frozen in liquid nitrogen. Tumors were classified according to the van Unnik grading system [27].

**Table 1:** Histopathological and clinical data in soft tissue sarcoma patients

	Serum OPN protein	Tumor OPN protein	Tumor OPN mRNA
Total	86	80	68
Tumor stage			
I	14	13	12
II	37	32	26
III	26	26	22
IV	9	9	8
Tumor type			
LS	21	21	18
FS and MFH	24	20	17
NS	8	7	3
RMS and LMS	22	22	20
Other STS	11	10	10
Irradiation			
Yes	31	32	29
No	55	48	39
Chemotherapy			
Yes	8	7	5
No	78	73	63
Tumor size			
T1	12	11	10
T2	74	69	58
Tumor resection			
Radical (R0)	57	54	49
Not Radical (R1)	29	26	19
Distant metastases			
M0	79	73	62
M1	7	7	6
Relapse			
Yes	55	51	47
No	31	29	21

Abbreviations: OPN: osteopontin, LS: liposarcoma, MFH: malignant fibrous histiocytoma; FS: fibrosarcoma; NS: neurogenic sarcoma; LMS: leiomyosarcoma; RMS: rhabdomyosarcoma; STS: soft tissue sarcoma;

### Reverse transcription-polymerase chain reaction and real-time quantitative PCR

Total RNA was isolated using the RNeasy Mini Kit according to the manufacturer's instructions (Qiagen, Hilden, Germany). Subsequently, 1000 ng of RNA was reverse transcribed with random hexamer primers and Superscript II reverse transcriptase (Invitrogen, Karlsruhe, Germany). The cDNA synthesis was performed as described previously [28]. The mRNA levels of OPN and the housekeeping gene, hypoxanthin phosphoribosyl-transferase (HPRT), were quantified using Quantitect SYBRGreen PCR kit (Qiagen, Hilden, Germany) in a Rotorgene 3000 thermal cycler (LTF Labortechnik, Wasserburg, Germany). The OPN and HPRT transcripts were amplified with primer pairs of Table 2. The PCR cycling conditions included incubation for 15 min at 95°C, followed by 40 cycles of 30 sec at 95°C, 30 sec at 60°C and 30 sec at 72°C. A melting curve analysis in a range from 65°C to 95°C (5°C/sec) was appended. OPN mRNA was quantified as molecules of OPN mRNA/pg HPRT mRNA.

**Table 2:** Primer sequences and primer locations applied for qRT-PCR

gene	primer	sequence 5' → 3'	Localization
HPRT	sense	5'-TTGCTGACCTGCTGGATTAC-3'	309-328
	antisense	5'-CTTGCGACCTTGACCATCTT-3'	551-570

OPN	sense	5'-TGGCCGAGGTGATAGTGTG-3'	555-573
	antisense	5'-CGGGGATGGCCTTGTATG-3'	686-703

### OPN-ELISA measurements

Tumor tissue extracts were prepared from frozen tissues using a standard extraction protocol. Briefly, 20 slices of 10 μm thickness were cut. The total protein was extracted by solubilizing the tissues in 1 ml Giordano buffer using sonication on ice. The cell lysates were clarified by centrifugation at 15000 rpm for 15 min. The protein content was determined using the Bradford assay. Fresh blood samples collected before surgical treatment were coagulated for 20 min at room temperature and centrifuged at 3000 rpm for 10 min (Sarstedt monovette, Nümbrecht, Germany). The serum and tumor tissue extracts were aliquoted and stored at -80°C until analysis. Aliquots of each sample were analyzed in duplicate using a commercial ELISA system for OPN (IBL International, Hamburg, Germany) according to the manufacturer's instructions [3].

### Statistical methods

All statistical analyses were performed with the SPSS software package 16.0 for Windows (SPSS Inc., Chicago, IL). The differences in the numerical data between two groups were evaluated using the Pearson's test or Fisher's exact test. For survival analyses the overall survival of STS patients was used as follow-up end point. The survival curves were generated using Kaplan-Meier analysis and the log rank test was used to test for differences. For univariate and multivariate analyses (adjusted to tumor stage) the Cox's proportional hazard regression model was used to calculate the hazard ratio in the analysis of survival. A p-value of less than 0.05 was considered to be statistically significant.

## Results

### Osteopontin levels in serum and tumor tissue

**Figure 1:**
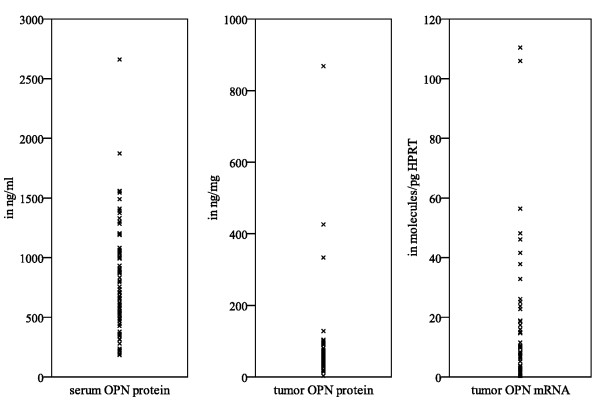
**OPN levels in serum and tumor tissue of STS patients**.

In patients with soft tissue sarcoma (STS) the median protein concentration of OPN in the serum and tumor tissue extracts was 704 ng/ml (184-2660 ng/ml) and 51.9 ng/mg (3.2-868 ng/mg), respectively (Fig. 1). The median OPN/HPRT transcript level ratio was 12.8 molecules OPN/pg HPRT (with a range of 0-110 molecules OPN/pg HPRT, Fig. 1). In particular, there was no correlation between OPN mRNA and OPN protein levels of tumors (n = 66; p = 0.4), serum and tumor OPN protein levels (n = 73; p = 0.9) or tumor OPN mRNA and serum OPN protein levels (n = 62; p = 0.7).

### Osteopontin levels and survival analyses

In subgroups split at the median serum or tumor OPN protein level, there was no significant difference in overall survival. However, when specimens were divided into four groups according to their OPN protein levels, the serum and tumor OPN protein levels did associate with different clinical parameters and overall survival. The group with the lowest tumor (≤ 34.34 ng/mg) or serum (≤ 516.54 ng/ml) OPN protein levels, i. e., the lowest quartile, had a favorable outcome compared to the other three groups (Table 3).

**Table 3:** Association of serum and tumor OPN levels with clinical parameters in soft tissue sarcoma patients

Category	Serum OPN protein level in ng/ml		Tumor OPN protein level in ng/mg		Tumor OPN mRNA level in molecules/pg HPRT	
	≤ 516.54	>516.54	≤ 34.34	>34.34	≤ 0.576	>0.576
Total	21	65	20	60	17	51
Tumor stage	p = 0.004*		p = 0.06		p = 0.21	
I	8	6	7	6	5	7
II	10	27	7	25	7	19
III	2	24	4	22	5	17
IV	1	8	2	7	0	8
Tumor type	p = 0.002*		p = 0.07		p = 0.19	
LS	12	9	10	11	5	13
FS+MFH	4	20	3	17	6	11
NS	0	8	2	5	2	1
RMS+LMS	3	19	4	18	3	17
Other STS	2	9	1	9	1	9
Tumor size	p = 0.03*		p = 0.85		p = 0.24	
T1	6	6	3	8	1	9
T2	15	59	17	52	16	42
Distant metastasis	p = 0.12		p = 0.82		p = 0.14	
M0	21	58	18	55	17	45
M1	0	7	2	5	0	6
Relapse	p = 0.41		p = 0.02*		p = 0.88	
R0	15	40	17	34	12	35
R1	6	25	3	26	5	16
Follow-up						
Alive	14	29	16	21	11	23
Dead	7	36	4	39	6	28
Kaplan-Meier analysis	p = 0.04*		p = 0.007*		p = 0.2	
mean survival in months	103	60	68	58	72	65
Cox's-Regression model	p < 0.05*		p = 0.01*		p = 0.21	
RR	2.2		3.7		1.7	
CI	1.0-5.1		1.3-10.4		0.7-4.3	

p* value was significant, when p < 0.05. Abbreviations: OPN: osteopontin, LS: liposarcoma, MFH: malignant fibrous histiocytoma; FS: fibrosarcoma; NS: neurogenic sarcoma; LMS: leiomyosarcoma; RMS: rhabdomyosarcoma; STS: soft tissue sarcoma; RR: relative risk of tumor-related death; CI: confidence interval of tumor-related death.

**Figure 2:**
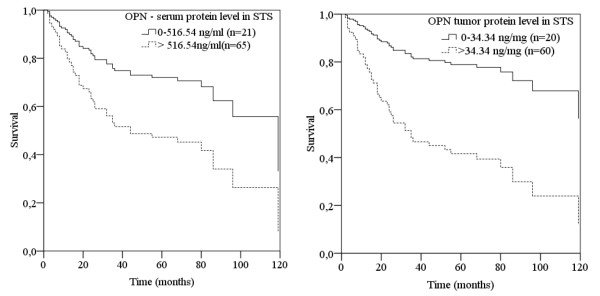
**Serum and tumor OPN protein levels and overall survival of STS patients**. Analysis of serum (left) and tumor (right) OPN protein levels and overall survival of STS patients using a univariate Cox's proportional hazard regression model. In comparison to patients with a low serum or tumor OPN-level (dotted line), an elevated OPN-level (bold line) correlates with a 2.2- (p < 0.05) or 3.7-fold (p = 0.01) increased risk of tumor-related death for STS patients.

Kaplan-Meier analyses showed a significant decrease of overall survival for the three groups with high OPN protein levels compared to the group with the lowest tumor (p = 0.007; log-rank test) and serum (p = 0.04; log-rank test) OPN protein levels. The mean survival time of the patient group with the lowest OPN protein level was 103 months (serum) and 68 months (tumor) whereas patients of the three remaining groups (high OPN protein levels) survived on average 60 months and 58 months (for serum and tumor tissue OPN protein levels, respectively). In addition, patients with both high serum and high tumor OPN protein levels had the worst prognosis (data not shown). Using a univariate Cox regression hazard model for STS patients with high OPN protein level in the serum or in tumor tissue extracts, the risk of tumor-related death was a 2.2 (95% confidence interval (CI) 1.01-5.1; p < 0.05) and 3.7 (95% confidence interval (CI) 1.3-10.4; p = 0.01), respectively (Fig. 2, Table 3). However, in a multivariate Cox's regression hazard model adjusted to tumor stage OPN protein level was not significantly correlated with prognosis.

### Bivariate analysis of osteopontin levels with clinical parameters

Bivariate analysis of OPN expression did show an association of serum and tumor OPN protein levels with different clinical parameters. In particular, high serum OPN protein levels correlated with high tumor stage (p = 0.004), grade of tumors (p = 0.003) and tumor size (p = 0.03). Additionally, all patients with early distant metastasis (7/86) had a high median serum OPN protein level of 1301 ng/ml (1009 to 2660 ng/ml) compared to patients without distant metastasis (79/86) who had a median serum OPN protein level of 657 ng/ml (183 to 1873 ng/ml). However, the difference was not significant (Table 3). A high tumor OPN protein level was significantly related to tumor grade (p = 0.003) and an increased rate of relapse (p = 0.02), and also showed a trend association with high tumor stage (p = 0.06) (Table 3). Furthermore, plasma OPN protein levels significantly correlated with tumor type (p = 0.002) whereas tumor OPN protein levels displayed only a trend association with tumor type (p = 0.07). Moreover, the patient group with the lowest serum and tumor OPN protein level was composed of 57% and 48% liposarcoma patients, respectively. In contrast, the three remaining patient groups with a high serum (>516.54 ng/ml) and tumor (>34.34 ng/mg) OPN protein level were composed of more than 80% of patients with malignant fibrous histiocytoma (MFH), fibrosarcoma (FS), leiomyosarcoma (LMS), rhabdomyosarcoma (RMS). In contrast to protein expression, statistical analysis of OPN mRNA levels showed no association with clinical parameters or clinical outcome (Table 3).

## Discussion

OPN overexpression is linked to an unfavorable prognosis in a variety of human cancers. However, only a few studies to date have investigated the prognostic impact of osteopontin in sarcoma patients. In the present study, we analyzed OPN mRNA and protein levels in tumors and the OPN protein levels from the serum of 93 soft tissue sarcoma patients and correlated OPN levels with clinical parameters and prognosis.

In our study no correlation was found between OPN levels in serum and tumor tissue. It is well known that extracellular OPN is involved to tumor progression with signaling to proliferation, migration and survival. Also intracellular OPN is involved in cell migration [29]. Additionally, there is also evidence that suggests that intracellular OPN has other cellular functions, particularly in relation to cell proliferation [30]. But regulation between intra- and extracellular OPN forms remains unknown. Nordsmark et al. (2007) assume a relation between plasma concentration of OPN and intratumoral OPN production [31]. However, in agreement with our results Nordsmark et al. (2007) detected no correlation between plasma OPN and tumor OPN protein levels in patients with head and neck cancer (p = 0.75). Furthermore, own in vitro studies detected a clear decrease of extracellular OPN protein levels after transfection with OPN siRNA in MDA-MB231 cells. In contrast, the intracellular OPN protein level was only partially decreased (Hahnel *et al*., manuscript submitted). More studies on the functions of intracellular OPN forms are necessary to understand their relationship with extracellular OPN and their tumor biological role.

Our data suggest that increased serum and tumor OPN protein levels are significantly associated with clinical parameters, such as tumor stage, tumor grade, subtype of tumor, tumor size and the rate of relapse (Table 3). In the Kaplan-Meier analysis, a significantly decreased overall survival was observed for patients with strong OPN protein expression in their serum (p = 0.04; Table 3) and tumor tissue (p = 0.007; Table 3). Furthermore, the univariate Cox's regression hazard model demonstrated that STS patients with a high serum and tumor OPN protein level have an increased risk of tumor-related death of 2.2 (p < 0.05) and 3.7 (p = 0.01; Fig. 2), respectively (Table 3). This is in agreement with a previously reported immunohistochemical study of 33 soft tissue sarcoma patients that showed an increased OPN level significantly correlated with higher tumor stage, grade and overall survival [22]. Furthermore, primary sarcomas of the pulmonary artery display an abundant immunohistochemical OPN protein staining of tumor cells and extracellular matrix. Therefore, OPN protein may play a substantial role in tumor progression of vessel sarcomas [21]. However, an immunohistochemical OPN study did not find any correlation between OPN protein expression and outcome of osteosarcoma patients [23]. In this regard, Luo et al. were able to show that different osteosarcoma cell lines had significantly lower OPN levels than mature osteoblasts [32]. This may indicate that OPN has no significance for osteosarcoma tumor growth. However, antisense oligodeoxynucleotides against human OPN reduced the tumorigenicity of xenotransplanted osteosarcoma tumors in nude mice [33]. Additionally, our analysis shows that siRNA-induced osteopontin inhibition results in reduced clonogenic survival and migration of soft tissue sarcoma and breast cancer cell lines (Hahnel *et al*., manuscript submitted).

Our analysis of tumor OPN mRNA expression of 68 soft tissue sarcoma patients did not show any association with clinical parameters or prognosis. In contrast, another study of 41 osteosarcoma patients did show that a high OPN mRNA level is correlative with overall survival, event-free survival and relapse-free survival [34]. Moreover, a study of 15 adult soft tissue sarcoma patients showed a significant increase of OPN mRNA levels compared to normal tissue [22]. In addition, OPN splice variants may be involved in tumor progression. In particular, osteopontin-c has been postulated to be a significant factor for glioma [35], breast cancer [17,36] and hepatocellular carcinoma [37]. Clearly, more data are needed to characterize the role of osteopontin mRNA in sarcomas.

## Conclusion

In the present study, we analyzed OPN mRNA and protein levels in tumors and the OPN protein levels from the serum of 93 soft tissue sarcoma patients. Our data suggest that increased serum and tumor OPN protein levels are significantly associated with clinical parameters, such as tumor stage, tumor grade, subtype of tumor, tumor size and the rate of relapse. In addition, using a univariate Cox's proportional hazards regression model, we found that an elevated OPN protein level in the serum and tumor tissue extracts is a significant negative prognostic factor for patients with STS. Our data suggest OPN protein in serum as well as in tumor tissue extracts is an important prognostic factor for soft tissue sarcoma patients.

## Competing interests

The authors declare that they have no competing interests.

## Authors' contributions

MB and DV designed the study, collected data, performed statistical analysis and drafted the manuscript. MKa, HW, SR, AH, TG, HMS and MKo and HT made substantial contributions acquisition of data, and analysis and interpretation of data. PW treated the patients, collected material and data and reviewed the manuscript. All authors read and approved the final manuscript.

## Pre-publication history

The pre-publication history for this paper can be accessed here:

http://www.biomedcentral.com/1471-2407/10/132/prepub

## References

[B1] Furger KA Menon RK Tuck AB Bramwell VH Chambers AF The functional and clinical roles of osteopontin in cancer and metastasis Curr Mol Med 2001 1 621 632 10.2174/1566524013363339 11899236

[B2] Bache M Kappler M Said HM Staab A Vordermark D Detection and specific targeting of hypoxic regions within solid tumors: current preclinical and clinical strategies Curr Med Chem 2008 15 322 338 10.2174/092986708783497391 18288988

[B3] Vordermark D Said HM Katzer A Kuhnt T Hänsgen G Dunst J Flentje M Bache M Plasma osteopontin levels in patients with head and neck cancer and cervix cancer are critically dependent on the choice of ELISA system BMC Cancer 2006 6 207 10.1186/1471-2407-6-207 16911785 PMC1564036

[B4] Wai PY Kuo PC Osteopontin: regulation in tumor metastasis Cancer Metastasis Rev 2008 27 103 118 10.1007/s10555-007-9104-9 18049863

[B5] Tuck AB O'Malley FP Singhal H Harris JF Tonkin KS Kerkvliet N Saad Z Doig GS Chambers AF Osteopontin expression in a group of lymph node negative breast cancer patients Int J Cancer 1998 79 502 508 10.1002/(SICI)1097-0215(19981023)79:5<502::AID-IJC10>3.0.CO;2-3 9761120

[B6] Rudland PS Platt-Higgins A El-Tanani M De Silva Rudland S Barraclough R Winstanley JH Howitt R West CR Prognostic significance of the metastasis-associated protein osteopontin in human breast cancer Cancer Res 2002 62 3417 3427 12067984

[B7] Boldrini L Donati V Dell'Omodarme M Prati MC Faviana P Camacci T Lucchi M Mussi A Santoro M Basolo F Fontanini G Prognostic significance of osteopontin expression in early-stage non-small-cell lung cancer Br J Cancer 2005 93 453 457 10.1038/sj.bjc.6602715 16091764 PMC2361587

[B8] Sakaguchi H Fujimoto J Hong BL Tamaya T Clinical implications of osteopontin in metastatic lesions of uterine cervical cancers Cancer Let 2007 247 98 102 10.1016/j.canlet.2006.03.026 16675104

[B9] Cho H Hong SW Oh YJ Kim MA Kang ES Lee JM Kim SW Kim SH Kim JH Kim YT Lee K Clinical significance of osteopontin expression in cervical cancer J Cancer Res Clin Oncol 2008 134 909 917 10.1007/s00432-007-0351-5 18210151 PMC12160727

[B10] Forootan SS Foster CS Aachi VR Adamson J Smith PH Lin K Ke Y Prognostic significance of osteopontin expression in human prostate cancer Int J Cancer 2006 118 2255 2261 10.1002/ijc.21619 16331611

[B11] Bache M Reddemann R Said HM Holzhausen HJ Taubert H Becker A Kuhnt Tm Hänsgen G Dunst J Vordermark D Immunohistochemical detection of osteopontin in advanced head-and-neck cancer: prognostic role and correlation with oxygen electrode measurements, hypoxia-inducible-factor-1alpha-related markers, and hemoglobin levels Int J Radiat Oncol Biol Phys 2006 66 1481 1487 17056190 10.1016/j.ijrobp.2006.07.1376

[B12] Celetti A Testa D Staibano S Merolla F Guarino V Castellone MD Iovine R Mansueto G Somma P De Rosa G Galli V Melillo RM Santoro M Overexpression of the cytokine osteopontin identifies aggressive laryngeal squamous cell carcinomas and enhances carcinoma cell proliferation and invasiveness Clin Cancer Res 2005 11 8019 8027 10.1158/1078-0432.CCR-05-0641 16299231

[B13] Chien CY Su CY Chuang HC Fang FM Huang HY Chen CM Chen CH Huang CC Clinical significance of osteopontin expression in T1 and T2 tongue cancers Head Neck 2008 30 776 781 10.1002/hed.20783 18228527

[B14] Schneider S Yochim J Brabender J Uchida K Danenberg KD Metzger R Schneider PM Salonga D Hölscher AH Danenberg PV Osteopontin but not osteonectin messenger RNA expression is a prognostic marker in curatively resected non-small cell lung cancer Clin Cancer Res 2004 10 1588 1596 10.1158/1078-0432.CCR-0565-3 15014008

[B15] Kolb A Kleeff J Guweidhi A Esposito I Giese NA Adwan H Giese T Büchler MW Berger MR Friess H Osteopontin influences the invasiveness of pancreatic cancer cells and is increased in neoplastic and inflammatory conditions Cancer Biol Ther 2005 4 740 746 15970685 10.4161/cbt.4.7.1821

[B16] Higashiyama M Ito T Tanaka E Shimada Y Prognostic significance of osteopontin expression in human gastric carcinoma Ann Surg Oncol 2007 14 3419 3427 10.1245/s10434-007-9564-8 17896150

[B17] Mirza M Shaughnessy E Hurley JK Vanpatten KA Pestano GA He B Weber GF Osteopontin-c is a selective marker of breast cancer Int J Cancer 2008 122 889 897 10.1002/ijc.23204 17960616

[B18] Patani N Jouhra F Jiang W Mokbel K Osteopontin expression profiles predict pathological and clinical outcome in breast cancer Anticancer Res 2008 28 6B 4105 4110 19192668

[B19] Weber GF The metastasis gene osteopontin: a candidate target for cancer therapy Biochim Biophys Acta 2001 1552 61 85 11825687 10.1016/s0304-419x(01)00037-3

[B20] Jain S Chakraborty G Bulbule A Kaur R Kundu GC Osteopontin: an emerging therapeutic target for anticancer therapy Expert Opin Ther Targets 2007 11 81 90 10.1517/14728222.11.1.81 17150036

[B21] Gaumann A Petrow P Mentzel T Mayer E Dahm M Otto M Kirkpatrick CJ Kriegsmann J Osteopontin expression in primary sarcomas of the pulmonary artery Virchows Arch 2001 439 668 674 11764388 10.1007/s004280100452

[B22] Bramwell VH Tuck AB Wilson SM Stitt LW Cherian AK Rorke SC Al-Katib W Postenka CO Chambers AF Expression of osteopontin and HGF/met in adult soft tissue tumors Cancer Biol Ther 2005 4 1336 1341 16258259 10.4161/cbt.4.12.2166

[B23] Sulzbacher I Birner P Trieb K Lang S Chott A Expression of osteopontin and vascular endothelial growth factor in benign and malignant bone tumors Virchows Arch 2002 441 345 349 10.1007/s00428-002-0671-4 12404059

[B24] Würl P Fittkau M Meye A Bartel F Schmidt H Schönfelder M Taubert H Low detection rate of p53 antibodies in sera of soft tissue sarcoma patients Cancer Lett 2001 170 199 205 10.1016/S0304-3835(01)00604-8 11463499

[B25] Würl P Kappler M Meye A Bartel F Köhler T Lautenschläger C Bache M Schmidt H Taubert H Co-expression of survivin and TERT and risk of tumour-related death in patients with soft-tissue sarcoma Lancet 2002 359 943 945 10.1016/S0140-6736(02)07990-4 11918915

[B26] Taubert H Würl P Greither T Kappler M Bache M Bartel F Kehlen A Lautenschläger C Harris LC Kaushal D Füssel S Meye A Böhnke A Schmidt H Holzhausen HJ Hauptmann S Stem cell-associated genes are extremely poor prognostic factors for soft-tissue sarcoma patients Oncogene 2007 26 7170 7174 10.1038/sj.onc.1210530 17525744

[B27] van Unnik JA Coindre JM Contesso C Albus-Lutter CE Schiodt T Sylvester R Thomas D Bramwell V Mouridsen HT Grading of Soft Tissue Sarcomas: Experience of the EORTC Soft and Bone Sarcoma Group Eur J Cancer 1993 29 2089 2093 10.1016/0959-8049(93)90039-I 8297645

[B28] Kappler M Kotzsch M Bartel F Füssel S Lautenschläger C Schmidt U Würl P Bache M Schmidt H Taubert H Meye A Elevated expression level of survivin protein in soft-tissue sarcomas is a strong independent predictor of survival Clin Cancer Res 2003 9 1098 1104 12631613

[B29] Zohar R Suzuki N Suzuki K Arora P Glogauer M McCulloch CA Sodek J Intracellular osteopontin is an integral component of the CD44-ERM complex involved in cell migration J Cell Physiol 2000 184 118 30 10.1002/(SICI)1097-4652(200007)184:1<118::AID-JCP13>3.0.CO;2-Y 10825241

[B30] Junaid A Moon MC Harding GE Zahradka P Osteopontin localizes to the nucleus of 293 cells and associates with polo-like kinase-1 Am J Physiol Cell Physiol 2007 292 C919 26 10.1152/ajpcell.00477.2006 17005603

[B31] Nordsmark M Eriksen JG Gebski V Alsner J Horsman MR Overgaard J Differential risk assessments from five hypoxia specific assays: The basis for biologically adapted individualized radiotherapy in advanced head and neck cancer patients Radiother Oncol 2007 83 389 397 10.1016/j.radonc.2007.04.021 17499868

[B32] Luo X Chen J Song WX Tang N Luo J Deng ZL Sharff KA He G Bi Y He BC Bennett E Huang J Kang Q Jiang W Su Y Zhu GH Yin H He Y Wang Y Souris JS Chen L Zuo GW Montag AG Reid RR Haydon RC Luu HH He TC Osteogenic BMPs promote tumor growth of human osteosarcomas that harbor differentiation defects Lab Invest 2008 88 1264 1277 10.1038/labinvest.2008.98 18838962 PMC9901484

[B33] Liu SJ Zhang DQ Sui XM Zhang L Cai ZW Sun LQ Liu YJ Xue Y Hu GF The inhibition of in vivo tumorigenesis of osteosarcoma (OS)-732 cells by antisense human osteopontin RNA Cell Mol Biol Lett 2008 13 11 19 10.2478/s11658-007-0031-0 17952379 PMC6275891

[B34] Dalla-Torre CA Yoshimoto M Lee CH Joshua AM de Toledo SR Petrilli AS Andrade JA Chilton-MacNeill S Zielenska M Squire JA Effects of THBS3, SPARC and SPP1 expression on biological behavior and survival in patients with osteosarcoma BMC Cancer 2006 6 237 10.1186/1471-2407-6-237 17022822 PMC1609181

[B35] Saitoh Y Kuratsu J Takeshima H Yamamoto S Ushio Y Expression of osteopontin in human glioma. Its correlation with the malignancy Lab Invest 1995 72 55 63 7837791

[B36] He B Mirza M Weber GF An osteopontin splice variant induces anchorage independence in human breast cancer cells Oncogene 2006 25 2192 2202 10.1038/sj.onc.1209248 16288209

[B37] Takafuji V Forgues M Unsworth E Goldsmith P Wang XW An osteopontin fragment is essential for tumor cell invasion in hepatocellular carcinoma Oncogene 2007 26 6361 6371 10.1038/sj.onc.1210463 17452979

